# Solution composition dependent Soret coefficient using commercial MicroScale Thermophoresis instrument†

**DOI:** 10.1039/d3ra00154g

**Published:** 2023-05-30

**Authors:** Praneetha Pulyala, Meng Jing, Wei Gao, Xuanhong Cheng

**Affiliations:** a Department of Bioengineering, Lehigh University Bethlehem PA 18015 USA xuc207@lehigh.edu; b Analytical Sciences, Core R&D, The Dow Chemical Company 400 Arcola Road Collegeville PA 19426 USA; c Department of Materials Science and Engineering, Lehigh University Bethlehem PA 18015 USA

## Abstract

Thermal diffusion of particles in dilute aqueous suspensions is driven by the interactions between the dispersing medium and the particle, which are largely influenced by the properties of the medium. Using a commercial instrument to generate thermophoresis, we developed a method to quantify the migration of colloids in a temperature gradient and further studied how it varies based on the composition and pH of the dispersing medium and with an anionic surfactant, at different salt concentrations. Thermophoretic migration of aqueous suspensions of carboxylate-modified polystyrene particles with different compositions is measured as MicroScale Thermophoresis (MST) traces and a mathematical model is developed to extract the Soret coefficient (*S*_T_). Soret coefficient measurements obtained using the developed method are in-line with previous theories and scientific findings from other literature, indicating a dependence of the *S*_T_ on the Debye length and surface charge density of the suspended particles, both of which are controlled by the composition of the dispersing medium. The thermophobic/thermophilic behavior of particles is also found to be strongly influenced by the thermoelectric effect of the buffer ions. In this paper, a new analytical model is introduced and applied to complex systems to understand their thermophoretic behavior as a function of solvent properties.

## Abstract 2

The developed method quantifies the thermophoretic migration of colloidal particles as the Soret coefficient, indicating a dependence on interfacial properties and ionic composition of the dispersing medium.

## Graphical abstract

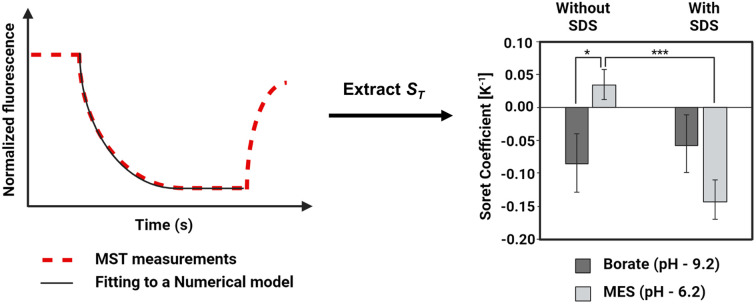

## Introduction

Thermophoresis, also known as thermodiffusion, is the directional migration of particles induced by a temperature gradient^1^ and is typically characterized by the *S*_T_. First observed by Carl Ludwig, this phenomenon has been studied in various systems including ions, aerosol particles, gases, and polymers.^2–6^ Most recently, thermodiffusion is gaining interest and has been studied extensively due to its applications. For instance, it is used to separate polymers and particles in field-flow fractionation,^7–9^ in combination with microfluidics for sub-micron particle sorting,^10–12^ and to monitor the protein-ligand binding in the pharmaceutical industry.^13–15^ A deeper understanding of the thermophoretic drift of colloidal particles could be used to manipulate particle migration, such as trapping DNA and vesicles for their diagnostic potential.

Traditionally, thermophoresis in aqueous suspensions has been experimentally studied using optical techniques such as beam deflection in a diffusion cell, forced Rayleigh light scattering, and the thermal lens method.^16–19^ Each of these methods has certain limitations such as interference from convection and long equilibrium time.^20,21^ In 2006, Duhr and Braun developed an all-optical fluorescence microfluidic technique that allows the measurement of thermodiffusion of colloid suspensions without the artifacts of convection.^22,23^ An IR-laser is used in this instrument to generate a temperature gradient in a thin film sample and the resulting fluorescence distribution is recorded as a function of time to monitor the migration of target species in the aqueous suspension. Initially developed to measure the thermodiffusion of particles and molecules, the instrument is now commercially available as the Monolith system, an analytical tool to monitor binding reactions of biomolecules under the influence of a temperature gradient. In this instrument, thermodiffusion of highly dilute suspensions can be measured in less than 1 minute with a low sample volume of 10 μL and complex fluid mixtures.^24,25^

Using the aforementioned optical techniques, a host of experimental investigations were performed with aqueous suspensions of colloids in the past few decades to evaluate the dependence of *S*_T_ on particle concentration, size, and surface coating, as well as the dispersing medium composition such as salinity and pH. Suspension of polystyrene (PS) particles is used as the model system in many of these studies.^26–30^ However, a gap persists between the experimental observations and theoretical explanations for the dependence of *S*_T_ on the properties of the dispersing medium. Theoretical models describe particle thermophoresis in a suspension as an interfacial phenomenon, driven by the interactions between the particle and the medium.^1,19^ Ruckenstein first described the thermophoretic motion as a result of the variation of interfacial tension at the particle–medium interface and derived an expression for a single particle *S*_T_ considering the analogy between the ‘phoretic’ motion of particles and the Marangoni effect of droplets.^31^ For the particle within the Debye Huckel regime, Duhr and Braun derived the most straightforward theoretical expression for *S*_T_ based on the solvation entropy and ionic shielding.^32^ They further experimentally validated this theory to predict *S*_T_ of PS and DNA in buffer solutions. Later, Dhont derived an explicit expression for the thermal diffusion coefficient in terms of the surface charge density of the particle, Debye length, and particle radius.^33^ Over a decade ago, Wurgur derived the thermal diffusion coefficient of particles based on the force density induced in the vicinity of the charged surface by the thermal gradient.^34^ Despite these complementing theories, there is still a need for quantification of migration due to thermal diffusion. Soret coefficient is an ideal candidate for quantitatively expressing this migration, however there is a need for a more consistent and robust method for a reproducible measurement.

In this work, we develop a method to quantify the thermophoretic migration of nanoparticles using the commercial MST instrument, Monolith NT.115. Key advantages of this instrument are the low sample volume requirement, faster equilibrium time and high detection sensitivity, which allow us to work with highly dilute solutions and avoid artifacts from inter-particle interactions. Fluorescence signals measured using this instrument are then analyzed using a numerical model to extract the thermophoretic characteristic of colloid particles as *S*_T_. The approach is first validated using PS particles with known *S*_T_ from previous work.^32^ Subsequently, we studied the dependence of *S*_T_ of carboxylate modified polystyrene (CPS) particles on buffer ionic concentration, pH of the solvent, and the presence of surfactant. The results are discussed in light of the literature and theoretical models. This novel combination of commercial instrument and analytical approach can be used to quantify thermophoretic particle migration for a variety of complex fluid mixtures by leveraging a consistent temperature gradient and theoretical validation.

## Materials and sample preparation

Aqueous suspensions of PS beads (Dyed Red Aqueous Fluorescent Particles, Thermo Scientific, Waltham, MA) were diluted from 1.0% (w/v) to 0.1% in deionized water for measurements. These beads were fluorescently labeled with Firefli Fluorescent Red, a proprietary fluorophore. The diameters of the beads are 0.02, 0.1, and 0.2 μm.

An aqueous suspension of 0.2 μm CPS beads (Fluoresbrite® YG Carboxylate Microspheres, Polysciences, Warrington, PA) was diluted from 2.60% (w/v) to 0.01% in 1, 10, and 100 mM borate buffer (pH = 9.2) and 2-morpholin-4-ylethanesulfonic acid (MES) buffer (pH = 6.2). The samples were then measured as-is or dialyzed using Float-A-Lyzer G2 dialysis devices (8–10 K MWCO, Repligen, Waltham, MA) against the respective buffer solution to remove additives in the original samples that might influence the thermophoretic properties of the suspensions. The dialysis was performed for 24 hours with the dialysate changed 3 times during the process. To study the influence of surfactant, an anionic surfactant, sodium dodecyl sulfate (SDS) (≥98.5%, Sigma Aldrich, St. Louis, MI) was added to the dialyzed CPS particles at a final concentration of 8.2 mM, the critical micellar concentration (CMC) of SDS in water.

## Method

### Surface charge density measurements

The surface charge density of the particles was characterized using a commercial Zetasizer Nano ZS (ZEN3600, Malvern Panalytical, Malvern, United Kingdom) at 25 °C. The samples were prepared in the same way as they were prepared to measure thermodiffusion. The surface charge of the particles in different buffer compositions was calculated using the Debye–Huckel equation. For moderate surface potential, where the Debye–Huckel approximation is valid, zeta potential *ζ* can be expressed as a function of surface charge *Q* as1
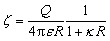
where *ε* is the dielectric constant, *R* is radius of the particle, and *κ* is the reciprocal of Debye length.^35^

### Measurement of MST traces

**Fig. 1:**
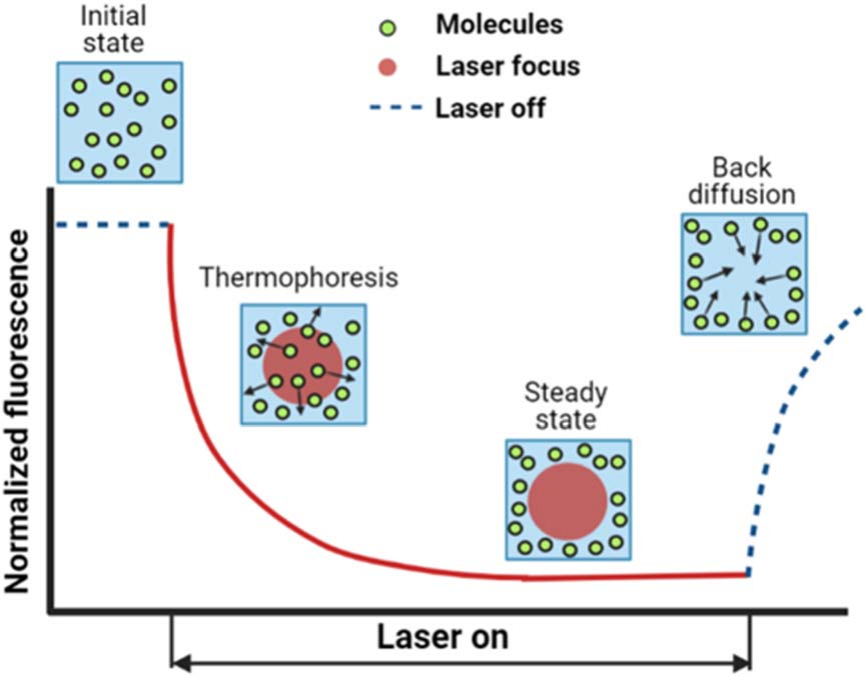
A typical MST trace recording the fluorescence intensity change over time upon laser exposure, adapted from ref. ^20^. Initially, the fluorescent colloids are homogeneously distributed, and a constant initial fluorescence is measured. As soon as the IR-laser is turned on, a fast fluorescence intensity decay is observed, due to TRIC, photobleaching and thermophoresis. After the IR-laser is switched off, the fluorescence intensity recovers.

The dynamic distribution of fluorescent particles in response to a temperature gradient was determined using MST measurements. The Monolith NT.115 instrument (Nanotemper Technologies, München, Germany) focuses an IR-laser with an emission wavelength of 1480 nm onto glass capillaries containing ∼10 μL samples, generating a microscale temperature gradient. Thermodiffusion of fluorescently labeled particles was measured dynamically as MST traces. The MST trace of PS and Fluoresbrite® particles was measured using the red laser with an excitation wavelength of 650 nm and the blue laser with an excitation wavelength of 493 nm respectively. The excitation power was set to 1% of the medium power based on the fluorescence counts evaluated in pre-tests. A schematic showing an MST trace of the dynamic fluorescence intensity is presented in Fig. 1, depicting the phases of the initial state, signal decay in response to the laser, and back diffusion after the laser is turned off.

### Temperature profile and particle distribution modelling

**Fig. 2:**
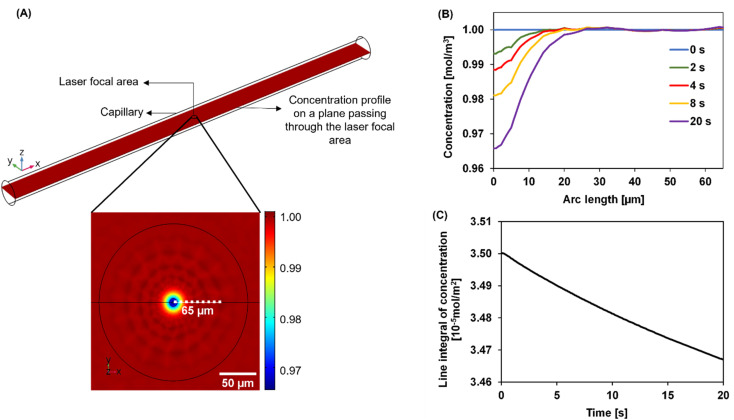
The simulation setup and typical results. (A, top) The simulation setup uses a laser focused on the center of the capillary to generate the temperature gradient. Here, parameters corresponding to 200 nm PS particles were used. The black circle indicates the center of the capillary on the plane passing through the laser focal area. (A, bottom) A zoom-in on the black circle shows the concentration depletion of the particles at the laser focal point along the radial temperature gradient. (B) Evolution of concentration from the center of the laser focus outwards at different time after the laser is switched on. While the temperature gradient is established within 1 s, the colloids deplete from the laser focal point gradually due to the slow mass transport process compared to heat transfer. (C) Line integral of concentration integrated over a line of 65 μm (dotted line in (A)). The concentration is noticed to decay non-linearly with time.

We developed a numerical model using the COMSOL Multiphysics® software (COMSOL Inc., Burlington, MA) to understand the dynamics of the particle distribution in response to the temperature gradient. In our model, the Mass Transport Module that captures diffusion and thermophoresis was coupled with the Heat Transfer Module that captures the temperature profile. A geometry similar to the experimental setup was used for the simulation (Fig. 2A). The laser, focusing at the center of a glass capillary of 0.2 mm radius, was described as a Gaussian beam with a narrow beam diameter of 10 μm. The heat load introduced by the laser on the sample was defined as shown in eqn (2).2
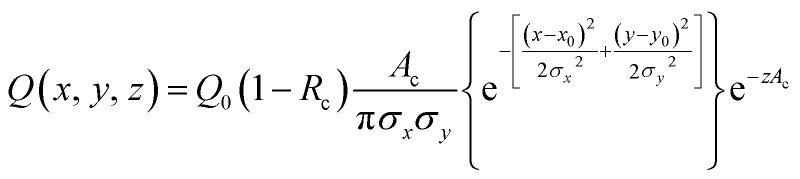


The reflection coefficient (*R*_c_) and absorption coefficient (*A*_c_) of the glass capillary within which the sample was present were considered 0.05 and 0.50 cm^−1^, respectively. *x*_0_ and *y*_0_ are the coordinates in the geometry corresponding to the center of the focused laser, and *σ*_*x*_, *σ*_*y*_ define the laser beam width. Laser input power *Q*_0_ was set to 0.05 W based on the MST power used for experiments. This laser source increases the temperature of the sample at the center of the focal region to ∼298 K, establishing a steady-state temperature gradient within 1 s of turning it on. The steady state temperature profile is shown in Fig. S1.† Using the previously established conditions, the COMSOL simulation solved for both the temperature gradient and a coefficient form partial differential equation (PDE) representing the particle migration. The representation of this coefficient form PDE was used to capture mass transport in response to the temperature gradient, requiring the simultaneous modeling of the two fluxes. The equation governing the returned coefficient form PDE can be seen in eqn (3).

3 *J* = −*D*∇*c* − *cD*_T_∇*T*

here, the initial concentration across the capillary was defined to be 1 mol m^−3^ and the diffusion coefficient *D* as 2.25 × 10^−12^ m^2^ s^−1^. The thermal diffusion coefficient *D*_T_ was calculated based on the defined *S*_T_ of the particles and temperature gradient ∇*T* was determined from the Heat Transfer Module. All other parameters in the simulation were also chosen to be a close approximation of the experimental parameters. Physical properties of water were used to define the material properties of the sample. A time-dependent concentration change in the capillary was simulated for 20 s, with the laser turning on at 0 s and off at 20 s, similar to the experimental MST measurements. Thermophoretic migration of particles with different *S*_T_ values were simulated using this model.

Over time, as the laser is turned on, the concentration profile shifts away from the uniformity seen at 0 s across the laser focal region wherein as the exposure to the laser increases, the particles migrate away from the focal region. In Fig. 2B, this effect is shown as the concentration decreases at the center of the focal region, especially as the time of laser exposure increases from 0 s to 20 s. The closer to the center, the more distinct the change in the concentration becomes with the laser exposure. As laser exposure time increases a more distinct temperature gradient is established, driving the particles farther from the focal point. Similarly, at 0 s there are more particles in the center of the focal region as the temperature gradient is initially being established. Over time, this concentration of particles in the focal region evolves even after the gradient is established due to the slow process of mass transfer compared to heat transfer. In Fig. 2C, there is a decrease in concentration over time-based on the movement of these particles out of the focal region in a non-linear fashion.

Mass change due to thermophoresis was analysed from COMSOL simulations in a disk-shaped volume with a planar diameter of 130 μm and disk thickness of 20 μm centred at the laser focal point. This disk is thicker than typical focal depth of microscopy lenses of 10× magnification or greater.^37^ The simulation results were compared against mass changes in a circular area. The 2D and 3D analyses yielded comparable results of relative mass change (Fig. S2†), thus usage of 2D integral results is still valid in curve fitting. We believe the comparable results in 2D and 3D are due to minor temperature variation in the axial direction of the disk than the planar direction, thus different *z*-planes in the disk have similar relative mass changes, leading to a cumulative 3D change comparable to changes in a 2D plane.

### Soret coefficient extraction from MST trace

The exponential decay of the fluorescence intensity in an MST trace (Fig. 1) upon switching on the laser results from multiple simultaneous effects occurring in the heated volume: colloid particles migration in response to the temperature gradient in addition to the decay phenomena in fluorophore such as temperature-related intensity change (TRIC) and photobleaching.

Decay phenomena resulting from the presence of the fluorophore need to be deconvoluted from the MST traces of the particles to extract the thermodiffusion properties of the particles, which is captured as *S*_T_. To develop this deconvolution procedure, suspensions of fluorescent polystyrene samples were dialyzed to extract the small amount of free fluorophore, whose MST traces were measured separately.

MST traces of the free fluorophore capture the decay due to TRIC and photobleaching, which are intrinsic to the fluorophore. MST traces from the particles on the other hand, do not follow a similar decay profile. In addition to the fluorophore decay, particle MST also captures the thermophoretic decay representing the particle migration. MST of the free fluorophore, representing decay in the fluorophore intensity has been characterized by an exponential function:^38^

4 Fluorophore intensity = *B* + *C* × e^−*c*×*t*^

where *B* is the background, *C* represents the initial fluorescence intensity of the fluorophore, and *c* represents the decay rate of the fluorophore with time *t*.

The MST traces of PS samples of known *S*_T_ were measured afterward. Assuming the decay rate of PS is the same as the fluorophore, the fluorescence decay of PS can be described as:

5 PS intensity = *D* + *E* × e^−*c*×*t*^ × Conc(*t*)

where the decay constant *c* extracted from the fluorophore measurement in eqn (4) was carried over to describe the fluorescence intensity decay of the particles. Here, *D* is the background and *E* is the initial intensity of the colloid sample. The product of e^−*c*×*t*^ and Conc(*t*) captures the composite effects that lead to the fluorescence intensity change, where the term Conc(*t*) represents the dynamic concentration of PS in the measurement volume due to colloid thermodiffusion.

## Results and discussion

**Fig. 3:**
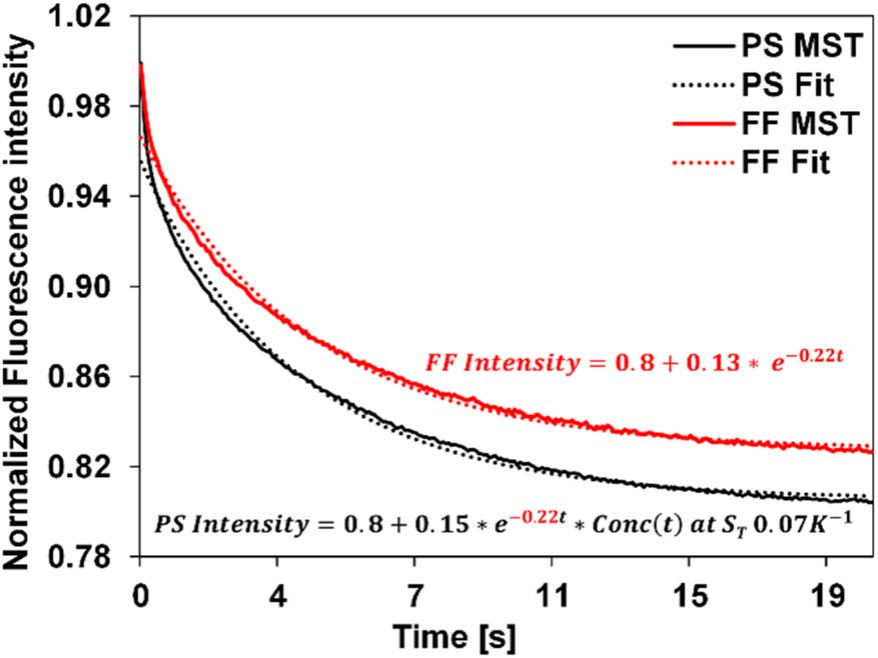
Experimental MST traces (solid) and the corresponding fit curves (dotted) for the fluorophore and the 20 nm PS, represented as FF and PS using eqn (4) and (5) respectively. The equation in red corresponds to the free fluorophore, whose decay constant is obtained by fitting the experimental fluorophore MST curve (*c* = −0.22) was carried over to fit the experimental PS curve as shown, resulting in the equation in black. Simulated Conc(*t*) using *S*_T_ = 0.07 K^−1^ best describes the 20 nm PS MST, leading to an extracted *S*_T_ of 0.07 K^−1^ for this PS measurement.

We applied the above-described method to determine the *S*_T_ values of colloid samples, Conc(*t*) was simulated corresponding to different *S*_T_ values and a custom data fitting program was used to identify the best fit to the experimental data using eqn (5). The Conc(*t*) in the best fit yields the sample *S*_T_. As an example, Fig. 3 presents the fitting of free fluorophore using eqn (4) and of PS using eqn (5). We first applied this fitting method to MST traces of 20 nm, 100 nm, and 200 nm PS samples of known *S*_T_ values. The extracted *S*_T_ shown in Table 1 are consistent with our previously reported values measured using other approaches.^36^

**Table 1:** Mean *S*_T_ predicted determined from MST traces for 20, 100 and 200 nm PS along with the standard deviations for a sample size of 8

PS size	*S*_T_ values from the literature^36^	Predicted *S*_T_
20 nm	0.05	0.07 ± 0.02
100 nm	0.18	0.17 ± 0.08
200 nm	0.66	0.60 ± 0.30

We then applied the MST approach to study the thermophoresis of commercial CPS samples. Specifically, the effects of ionic concentration and pH on particle migration at room temperature have been studied. The influence of an anionic surfactant, SDS on the thermophoretic migration of particles, has also been evaluated.

### Effect of ionic concentration

**Fig. 4:**
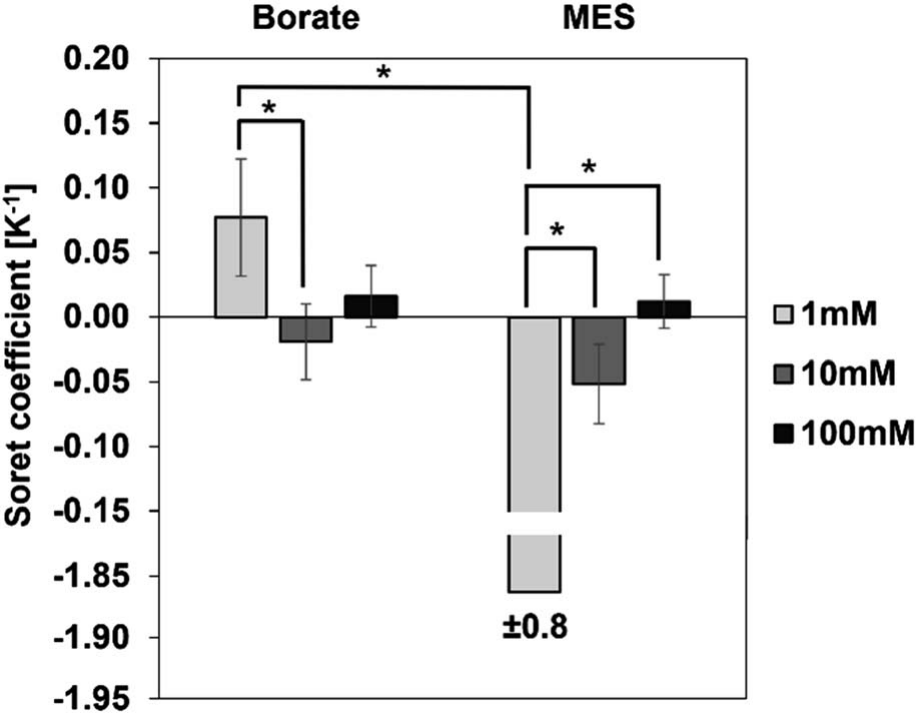
Measured *S*_T_ of as-received CPS in borate and MES buffers at ionic concentrations 1, 10 and 100 mM. In 1 mM borate buffer, particles exhibit a strong positive *S*_T_. At the same ionic concentration in MES, particles exhibit a strong negative *S*_T_. At 10 and 100 mM ionic concentrations in both buffers, the magnitude of *S*_T_ is close to zero and not significantly different from each other (*n* = 8).

We first investigated the thermophoretic properties of the CPS particles as a function of ionic concentration. Particle suspensions received from the supplier were diluted to 0.01% (w/v) in borate buffer (pH 9.2) and MES buffer (pH 6.2) at concentrations of 1, 10, and 100 mM. Fig. 4 represents the measured *S*_T_ of these particles as a function of ionic concentration. In both buffers, the magnitude of *S*_T_ was observed to decrease with increasing ionic concentration. For instance, in the case of borate buffer, with the ionic concentration increasing from 1 to 100 mM, the magnitude of *S*_T_ decreases from 0.08 ± 0.05 K^−1^ to approximately zero.

A similar trend is observed in the MES buffer, where the magnitude of *S*_T_ decreases from 1.86 ± 0.75 K^−1^ to approximately zero. *S*_T_ of particles in 10 mM buffer is not significantly different from the *S*_T_ in 100 mM buffer in both borate and MES buffers.

It was noticed that some samples present a large standard deviation. To verify measurement reproducibility, independent experiments have been carried out on the same sample composition and the MST traces are found to be reproducible across these experiments. As an example, MST traces of as-received CPS particles in 1 mM MES are shown in supplemental data (Fig. S3†). Another contribution to the large standard deviation is sample inhomogeneity. Sample inhomogeneity and particle aggregation can be identified by “bumpiness” or sudden jumps in the MST trace when an aggregate passes through the detection region. No such indications were observed in our measurements even for the samples where the extracted *S*_T_ have huge variations (Fig. S4†). Rather, we speculate that the large standard deviation in some Soret coefficient measurements is a result of dominating fluorescence decay of the fluorophore alone relative to the nanoparticle migration (Fig. S3†). Hence the extracted *S*_T_ has a relatively large variation even for fairly consistent measurements. Nonetheless, the particle thermophoresis is detectable and differentiable for different experimental conditions using the developed method.

Ionic concentration of the dispersing medium plays a critical role in the thermodiffusion of particles, as the concentration of ions alters the Debye length *via* the surface shielding phenomenon. The Debye length is inversely proportional to the ionic concentration, *i.e.*, the Debye length is small at higher ionic concentration of the dispersing medium. At a buffer concentration of 100 mM, oppositely charged species effectively shield the particle surface, reducing the Debye length significantly. As observed in Fig. 4, the magnitude of *S*_T_ is the greatest at 1 mM buffer concentration, and it diminishes to approximately zero at 100 mM. In other words, particles exhibit stronger thermal migration characteristics at lower ionic concentrations, where the surface shielding is less and hence a larger Debye length. Theoretical models developed by Ruckenstein,^31^ Dhont,^33^ and Wurger^34^ present explicit expressions for the contribution of the electrical double layer to the *S*_T_ of suspended particles. Based on these theories, *S*_T_ has a quadratic dependence on the surface charge density of the particles and scales linearly with the Debye length:

6 *S*_T_ ∝ *σ*^2^*λ*

where *σ* and *λ* are surface charge density and Debye length of the particle. Such a dependence of *S*_T_ on the Debye length has been validated experimentally by many researchers.^26,32,39,40^ Specifically, Duhr and Braun confirmed the contributions from surface charge density and Debye length to the *S*_T_ of CPS particles and DNA at different ionic concentrations. Within the range of ionic concentrations evaluated in this work, the surface charge density of the CPS particles can be considered virtually independent of the ionic concentrations, making *S*_T_ linearly proportional to the Debye length of the particles.^32,41^

Since particle thermodiffusion is most prominent in 1 mM buffer solutions, we further studied the effect of pH and surfactant on the particle *S*_T_ at this concentration. However, Fig. 4 was generated using commercial samples as is, and proprietary additives in the suspensions make it difficult to interpret the sign change in the two different buffers. Thus, particle suspensions were dialyzed against 1 mM borate or MES buffers to remove soluble additives and control the composition, enabling the correlation of experimental data with existing theory in the literature. These two buffers control the solution pH at 9.2 and 6.2 respectively. At both pH conditions, we also evaluated the effect of surfactant on *S*_T_ using an anionic surfactant SDS. Results with dialyzed samples are described below.

### Effect of pH and components in the dispersing medium

**Fig. 5:**
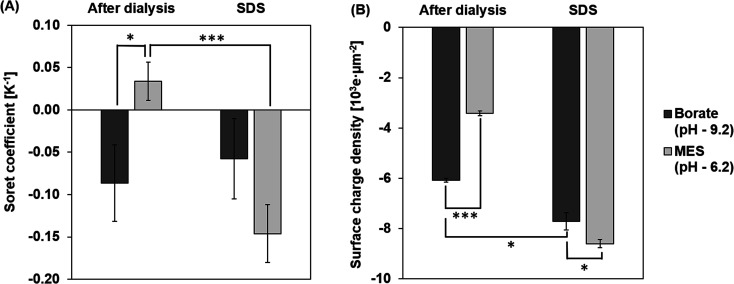
Measured (A) *S*_T_ and (B) surface charge density of CPS particles after dialysis and after addition of SDS in 1 mM borate and MES buffers. The samples after dialysis are referred to as AD and samples with subsequently added surfactant are referred to as SDS.

*S*_T_ measurements of the particles at pH 9.2 and 6.2 after dialysis are shown in Fig. 5A. The magnitude of *S*_T_ is higher for particles in borate, at a pH 9.2 compared to particles in MES buffer at pH 6.2. At a pH of 9.2, the charge density increases due to the deprotonation of carboxyl groups on the surface of the particles.^42^ Particles are less deprotonated in the MES buffer at a pH of 6.2 and hence the surface charge density on the PS particle is lower in the MES buffer. This effect of pH on the surface charge density of the particle is captured in Fig. 5B. Based on the dependence of *S*_T_ on the surface charge density presented in eqn (6), particles in the borate buffer are expected to have a higher magnitude of *S*_T_ than the particles in the MES buffer.^42^ In addition to this observation, we also notice a sign change in *S*_T_ due to the composition change of the dispersing medium. Particles in MES after dialysis have a positive *S*_T_ or “thermophobic” behavior. *S*_T_ of particles changes to negative or “thermophilic” in borate buffer. Such a change in the sign of *S*_T_ values could be explained by the thermoelectric effect induced by the ions in the dispersing medium.^43,44^ It is understood that electrolytes in the dispersing medium are subjected to thermodiffusion and anions have a stronger tendency to accumulate towards the cold side than the cations, generating a thermoelectric field that repels negatively charged particles from the cold side. The thermophoretic particle migration is hence a result of the competing motions between the anions and the negatively charged particles accumulating on the cold side. A strong thermoelectric field from the ions is expected to counteract the thermophobic response of the particles, leading to thermophilic migration and negative *S*_T_ of the particles.^42,45,46^ A reversal of the sign of *S*_T_ has been noted for particles in NaOH and NaCl from negative to positive, attributing to a stronger thermal response of OH^−^ ions than Cl^−^ ions.^45^ Since the cations in the two tested buffers are the same (Na^+^), the thermoelectric effect is controlled by the anions in the borate and MES buffers. Albeit no literature has been found about the thermodiffusion of borate and MES anions, the opposite signs of *S*_T_ of CPS in these two buffers support that the thermoelectric effect in the borate buffer is much stronger than that in the MES buffer.

### Effect of surfactant

Surfactants, often employed to stabilize colloid particles in a suspension, adsorb on the particle surface and alter the particle–solvent interface. As thermophoresis is strongly dependent on interfacial characteristics, the *S*_T_ of the colloidal samples could be significantly altered by surfactants. In this work, an anionic surfactant, SDS is added to the dialyzed samples at its CMC of 8.2 mM. Fig. 5A compares the measured *S*_T_ of particles with and without SDS in borate and MES buffers.

After adding SDS, *S*_T_ values of the particles are negative (thermophilic) regardless of the pH and anion species. The *S*_T_ of CPS in borate and MES buffers with SDS are −0.06 ± 0.05 K^−1^ and −0.15 ± 0.03 K^−1^, respectively. As discussed before, the sign and magnitude of *S*_T_ are controlled by the surface charge density and medium composition, while the dominant anion species in the SDS medium is the dodecyl sulfate ion, considering the SDS concentration of 8.2 mM and buffer ion concentration of 1 mM. Thus, the *S*_T_ of CPS in the two buffers is expected to have the same sign due to the comparable thermoelectric effect. In addition, SDS absorbed on the surface of CPS particles masks the native carboxyl chemistry, leading to thermomigration governed by the surfactant coating rather than the intrinsic surface chemistry of the particles.^10,26^ Thus, the magnitude of particle *S*_T_ is mostly controlled by the surface charge density. As seen in Fig. 5B, particles in borate and MES buffers with SDS have relatively comparable surface charge densities of 7.7 × 10^3^ ± 0.3 × 10^3^ e μm^−2^ and 8.6 × 10^3^ ± 0.2 × 10^3^ e μm^−2^. This difference in the surface charge density leads to a greater magnitude of *S*_T_ in MES than in the borate buffer.

## Conclusion

We developed a novel combination method to quantify the thermodiffusion of nanoparticles in aqueous suspensions using measurements from a commercial MicroScale Thermophoresis instrument^14,25,47,48^ and a physics-informed fitting model. Together with the reliable temperature gradient, the key advantages of using the commercial instrument compared to the existing approaches are its low sample consumption, high reproducibility with the standard capillaries, and short equilibration time, circumventing local heating.^20,49–51^ Additionally, the data fitting approach employed here deconvolutes fluorophore photobleaching from the measured thermophoretic migration to extract purely particle migration characteristics.

Using polystyrene as model particles, we compared the measured Soret coefficient using the method developed in this work and found a reasonable agreement with the literature of experimental and theoretical predictions.^19,26,31,32,39,52–55^ Further, the measured Soret coefficient of carboxylate-modified polystyrene particles indicate a strong dependence on the solvent composition, including the ionic concentration and species, pH, and presence of surfactant molecules, confirming thermodiffusion is an interfacial phenomenon and is largely influenced by the properties of the medium.^42,45,46^ Observed results are in-line with the experimental and theoretical predictions for carboxylate-modified polystyrene particles, while examining more solution compositions and extending this characterization method to different particle types in the future will provide more insights about thermophoresis for different colloidal systems. The method can further be developed into a characterization technique to monitor the stability of colloids in a temperature gradient based on the measured Soret coefficient. Future work will also be directed towards studying the influence of interfacial properties on particle thermophoresis in complex colloidal systems to establish a correlation between the Soret coefficient and the stability of the system.

## Author contributions

Praneetha Pulyala: methodology, formal analysis, investigation, software, writing – original draft, visualization. Meng Jing: resources, conceptualization, writing – review & editing, supervision. Wei Gao: conceptulization, methodology, resources, supervision, funding acquisition. Xuanhong Cheng: conceptualization, writing – review & editing, supervision, funding acquisition.

## Conflicts of interest

There are no conflicts to declare.

## Supplementary Material

RA-013-D3RA00154G-s001

RA-013-D3RA00154G-s002
