# Supplementary material for: Solution composition dependent Soret coefficient using commercial MicroScale Thermophoresis instrument
Source: RSC Adv. 2023 May 30;13(23):15901–9. doi: 10.1039/d3ra00154g (PMC10228179; doi:10.1039/d3ra00154g)
Supplement: RA-013-D3RA00154G-s002 [file RA-013-D3RA00154G-s002.pdf]

Electronic Supplementary Material (ESI) for RSC Advances.
This journal is © The Royal Society of Chemistry 2023

Supplemental information for submission "Solution Composition Dependent Soret Coefficient Using Commercial MicroScale Thermophoresis Instrument" by Praneetha Pulyala, Meng Jing, Wei Gao, Xuanhong Cheng

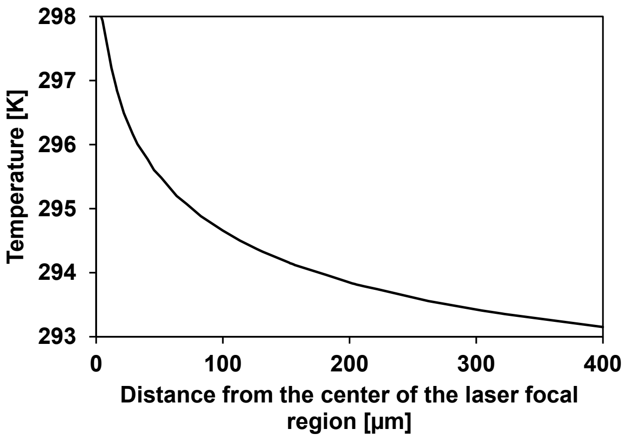

Fig. S1. Temperature profile along the radial direction from the laser focal region. Temperature gradient is steep within ~100µm and gradually decreases until the temperature at the boundary reaches room temperature.

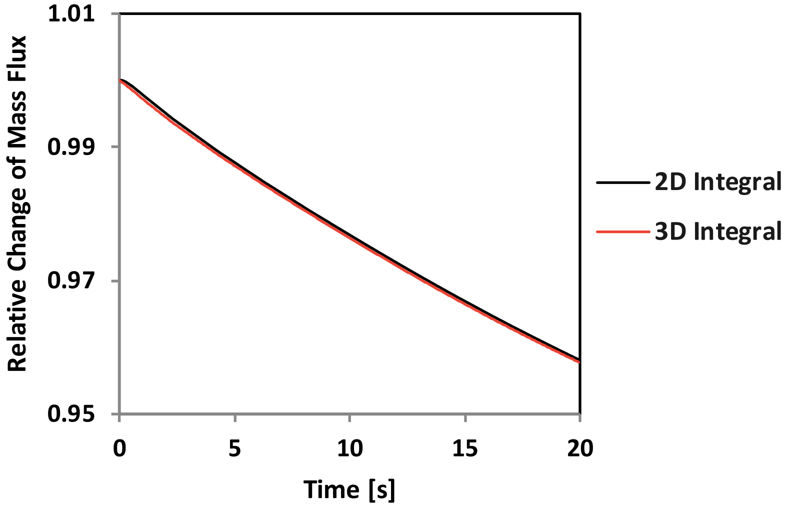

Fig. S2. Comparison of mass changes calculated by 2D vs. 3D integration of simulated concentration. For the 2D integration, the concentration was integrated over a circular area center of 130µm in diameter around the simulated laser focal point. 3D integration used a disk-shaped volume with a planar diameter of 130µm and disk thickness of 20µm. St = 0.6 in this simulation. The two integrals yielded comparable results of relative mass change, thus 2D integrals are used for curve fitting.

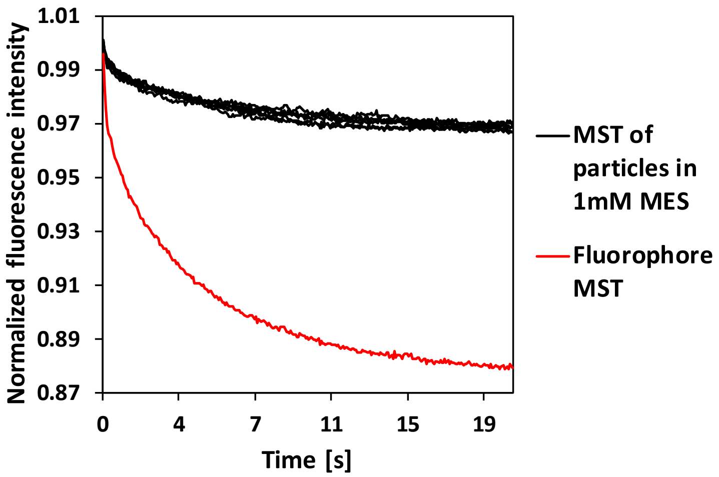

Fig. S3. Experimental MST traces of as received CPS particles in 1mM MES in comparison with the fluorophore MST. Reproducibility of the data has been verified by performing independent preparation and measurements of the same composition, and the MST traces are found to be reproducible as indicated by the black lines. The fluorescence decay of the fluorophore, relative to the CPS particle migration is dominant resulting in a large standard deviation in the Soret coefficient extracted for this experimental condition.

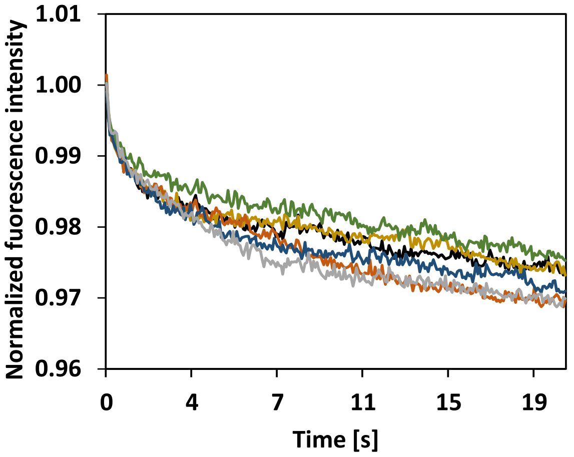

Fig. S4. Repeated measurements of MST traces of CPS particles in 1mM borate buffer after dialysis. Extracted Soret coefficient for this experimental condition have an average of -0.09 and standard deviation of 0.05, while measured MST traces do not exhibit obvious features indicating aggregates or sample inhomogeneity, which are indicated by "bumpiness" or sudden jumps in the trace[1,2].

References

1. Entzian, C. & Schubert, T. Studying small molecule–aptamer interactions using MicroScale Thermophoresis (MST). *Methods* **97**, 27–34 (2016).
2. Sedivy, A. Standard operating procedure for NanoTemper Monolith measurements. *European Biophysics Journal* **50**, 381–387 (2021).
